# Electrochemical detection of chloramphenicol using gadolinium tungstate with sulphur-doped carbon nitride nanocomposite

**DOI:** 10.5599/admet.3015

**Published:** 2025-12-08

**Authors:** Trishul Alanahally Mallu, Gagankumar Sakleshpur Kumar, Santhosh Arehalli Shivamurthy, Nalini Seetharamaiah, Manoj Kumar Basavarajappa, Sandeep Shadakshari

**Affiliations:** 1 Department of Environmental Engineering, SJCE, JSS Science and Technology University, Mysuru, Karnataka, India; 2 Department of Chemistry, SJCE, JSS Science and Technology University, Mysuru, Karnataka, India; 3 Department of Chemistry, NMKRV College for Women, Jayanagar, Bengaluru, Karnataka, India; 4 Independent Researcher, Bengaluru, Karnataka, India

**Keywords:** Electrochemical sensor, antibiotic detection, cyclic voltammetry, nanocomposite, pharmaceutical analysis

## Abstract

**Background and purpose:**

Chloramphenicol (CAP) is a broad-spectrum antibiotic whose unregulated presence in pharmaceuticals and food products raises significant health concerns, underscoring the need for rapid, reliable detection methods. This study aimed to develop a sensitive and economical electrochemical sensing platform based on a novel gadolinium tungstate (Gd_2_(WO_4_)_3_) and sulphur-doped graphitic carbon nitride (S-g-C_3_N_4_) nanocomposite for the efficient determination of CAP.

**Experimental approach:**

The Gd_2_(WO_4_)_3_/S-g-C_3_N_4_ nanocomposite was synthesized via a simple co-precipitation method and characterized using XRD, XPS, EDS, and TEM to confirm structural and morphological integration. A glassy carbon electrode modified with the composite was evaluated by cyclic and linear sweep voltammetry, along with analyses of interference, repeatability, stability, and real samples in eye-drop formulations and milk.

**Key results:**

The modified electrode exhibited significantly enhanced electrocatalytic oxidation of CAP compared with bare and individually modified electrodes, demonstrating high sensitivity, good selectivity against common interferents, and strong operational stability and reproducibility. A low detection limit was achieved, and the electrode effectively quantified CAP in real matrices with satisfactory recovery.

**Conclusion:**

The findings establish the Gd_2_(WO_4_)_3_/S-g-C_3_N_4_ nanocomposite as an efficient sensing material, offering a reliable, stable, and cost-effective platform for routine monitoring of antibiotic residues. While minor optimization may further expand its applicability, the study advances electrochemical sensing by introducing a robust nanocomposite with improved analytical performance for CAP detection.

## Introduction

Chloramphenicol (CAP), a widely known antibiotic with broad-spectrum antibacterial activity, has been used extensively to treat a variety of infectious diseases in humans and animals [1-3]. However, in recent years, CAP has also been known to take a toll on human health and has side effects that cause serious illness like aplastic anaemia [4], fatal bone marrow depression [5], agranulocytosis and gray baby syndrome [6,7]. In these lines, a few countries (including China, North America and Canada) have constrained the usage of CAP while European nations have set a maximum trace of CAP limit of 0.3 g kg^-1^ for food-safety control activities [8]. Nevertheless, it is still widely used in developing countries due to its efficiency, affordability, and availability. As a result, it is a necessary task to determine the concentration of CAP in the sample by using efficient, low-cost, rapid analytical techniques and examine their applicability in human fluids, food and drug samples [9]. Numerous microbiological and analytical techniques are presently available for CAP determination, in particular, liquid chromatography-tandem mass spectrometry (LC-MS/MS), gas chromatography-mass spectrometry (GC-MS), gas chromatography with electron capture detection (GC-ECD), radioimmunoassay, enzyme immunoassay [10], and capillary electrophoresis [11]. Although it is reliable and accurate, there are certain limitations related to its cost and complex operational procedures, which may limit the application of these techniques [12]. Hence, to overcome these drawbacks, the electrochemical technique has been widely used. The advantage of the electrochemical method includes very high sensitivity, rapid analysis, ease of use and low cost [13].

Recently, rare-earth minerals and their derivatives have emerged as the most promising choice for a variety of electrochemical sensors due to their superior intrinsic properties, such as high electrical conductivity and excellent magnetic, optical, and chemical properties [14]. Tungstates of rare earth metals like, Gd_2_(WO_4_)_3_, La_2_(WO_4_)_3_, Sm_2_(WO_4_)_3_, Ce_2_(WO_4_)_3_, Tm_2_(WO_4_)_3_ *etc.* have been thoroughly investigated over the previous years in the applications of sensors owing to low toxicity and high surface coverage area, high thermal and chemical stability [15-17]. These research studies suggest that gadolinium tungstate could be a promising candidate in the construction of an electrochemical sensor.

Similarly, it has been documented in the literature that altering electrodes with noble metal-free substances like graphene and graphitic carbon nitride (g-C_3_N_4_) enhances the electrode's catalytic performance. In addition to that, g-C_3_N_4_-based materials can be easily synthesised using low-cost precursors [18]. Furthermore, in a wide assortment of electrochemical sensing applications, g-C_3_N_4_ has been efficiently applied for the determination of biomolecules, phenolic compounds and toxic metal ions, attributing to its polymeric nature, making it a great fit for electrocatalytic materials [19]. Recently, in the field of carbon nanomaterials, doping g-C_3_N_4_ with heteroatoms like phosphorus, oxygen, boron, halogen, and sulphur has received a lot of attention. It is noteworthy that, especially the sulphur-doped bulk g-C_3_N_4_, even at modest concentrations, can be effectively tuned by leveraging its intrinsic properties, such as a high surface area, excellent chemical properties, and extraordinary electronic properties.

Both Gd_2_(WO_4_)_3_ and S-g-C_3_N_4_ are known for their unique electronic structures and excellent catalytic properties. When combined, these materials form a heterojunction interface between two semiconductors, which facilitates efficient charge separation and transfer. Previous studies on gadolinium-modified g-C_3_N_4_ and tungsten oxide (WO_3_) composites have demonstrated the formation of an S-scheme heterojunction, a mechanism that enhances redox efficiency and stability. This suggests that a similar interaction is likely to occur between Gd_2_(WO_4_)_3_ and S-g-C_3_N_4_. Moreover, composites of tungstate and doped g-C_3_N_4_ have shown remarkable potential in environmental remediation and energy-related applications such as photocatalytic degradation of organic pollutants, hydrogen evolution from water splitting, air purification, and antibacterial activity [20]. Motivated by these promising properties, we aimed to extend the application of this heterostructured nanocomposite to the field of electrochemical sensing. The synergistic effect between Gd_2_(WO_4_)_3_ and [S-g-C_3_N_4_] is expected to enhance electron transfer kinetics and improve the sensitivity and selectivity of CAP detection.

In the present research work, Gd_2_(WO_4_)_3_ was synthesized using a co-precipitation approach and S-g-C_3_N_4_ was prepared by the combustion process. The resultant Gd_2_(WO_4_)_3_/S-g-C_3_N_4_ nanocomposite was characterized using spectrophotometry and voltammetry techniques. Then, the nanocomposite was explored in the construction of an electrochemical sensor by adsorbing it onto a GCE for the reduction of CAP. The modified electrode exhibited an excellent linear range and a low limit of detection. Furthermore, the reproducibility, storage stability, and practicability were investigated.

## Experimental

### Chemicals and reagents

Gadolinium nitrate hexahydrate (Gd(NO_3_)_3_·6H_2_O), sodium tungstate dihydrate (Na_2_WO_4_·2H_2_O), melamine, and thiourea were procured from Sigma-Aldrich (Bangalore, India) and used without further purification. potassium ferricyanide (K_3_Fe(CN)_6_), potassium ferrocyanide (K_4_Fe(CN)_6_), potassium chloride (KCl), and phosphate-buffered saline (PBS, pH 7.0) were obtained from Merck (Mumbai, India). CAP standard solution was purchased from Himedia Laboratories Pvt. Ltd. (Mumbai, India). All aqueous solutions were prepared using double-distilled water. Electrochemical experiments, including cyclic voltammetry (CV) and electrochemical impedance spectroscopy (EIS), were performed using a CHI 6041E electrochemical workstation (CH Instruments, USA) with a conventional three-electrode system comprising a glassy carbon working electrode (3 mm diameter), a platinum wire counter electrode, and an Ag/AgCl (3 M KCl) reference electrode.

### Apparatus

The morphological structure and elemental mapping of the as-synthesised nanocomposite were examined using an EDX-equipped TEM (JEM 2100F; JEOL Ltd., Japan). The chemical composition and structural crystallinity were examined using XPS (JPS-9030, JEOL ltd., Japan) and XRD (X′ Pert3 Powder, Malvern Panalytical/UK) techniques. Electrochemical measurements were carried out with CHI 6041E electrochemical workstation, using a conventional three-electrode system where a saturated calomel electrode, a glassy carbon electrode and a platinum electrode were used as reference, working and auxiliary electrodes, respectively.

A glassy carbon electrode (GCE, 3 mm diameter) modified with the synthesized nanocomposite served as the working electrode, a platinum wire as the counter electrode, and an Ag/AgCl (3 M KCl) electrode as the reference. All experiments were performed in 0.1 M phosphate-buffered saline (PBS, pH 7.0) at room temperature (25±2 °C).

Cyclic voltammetry (CV) was conducted within a potential range of -0.4 V to +0.2 V at a scan rate of 50 mV s^-1^. The current response was measured at different concentrations of the target analyte (50-250 μM) under identical conditions. Electrochemical impedance spectroscopy (EIS) was performed at the open-circuit potential using a 5 mV AC amplitude over a frequency range of 0.01 Hz to 100 kHz. Nyquist plots were fitted with an equivalent circuit to determine the charge-transfer resistance (*R*_ct_) and evaluate electron-transfer characteristics.

### Synthesis of Gd_2_(WO_4_)_3_ and fabrication of Gd_2_(WO_4_)_3_/S-g-C_3_N_4_ nanocomposite

**Scheme 1.**
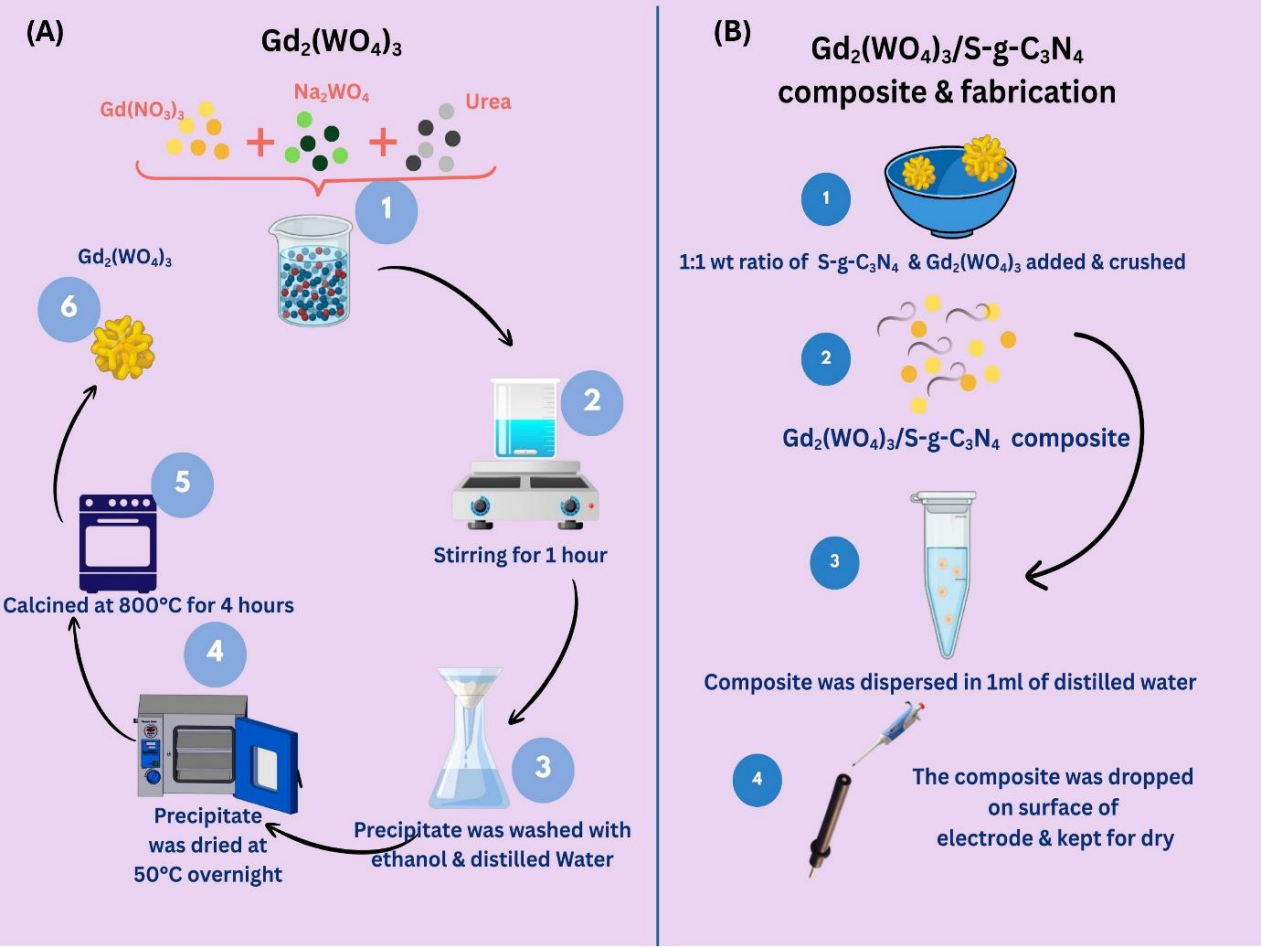
(A) Graphical representation of synthesis of Gd_2_(WO_4_)_3_/S-g-C_3_N_4_ composite and (B) fabrication of electrode

Gadolinium tungstate (Gd_2_(WO_4_)_3_) was synthesized using the co-precipitation method. 200 mL of distilled water was used to dissolve 0.1 M Gd (NO_3_)_3_ and 0.2 M Na_2_WO_4_. The morphological influence was improved by adding 0.5 M urea (0.6 g in 20 mL) to the solution and stirring continuously for 1 hour at room temperature, resulting in a white precipitate. Finally, the product was washed and thoroughly cleaned with ethanol, followed by deionised water. The precipitate was then dried at 50 °C overnight. The sample was then calcinated at 800 °C for 4 hours at a rate of 5 °C/min (Scheme 1A). Besides this, S-g-C_3_N_4_ was synthesized by pyrolyzing 10 g of thiourea (CH_4_N_2_S) in a muffle furnace at 540 °C for 5 h. A simple grinding process was then employed to create Gd_2_(WO_4_)_3_/S-g-C_3_N_4_ composite. Consequently, equal parts of Gd_2_(WO_4_)_3_ and S-g-C_3_ N_4_ (1:1 weight ratio) were mixed and crushed to a fine paste in a mortar (Scheme 1B).

### Fabrication of Gd_2_(WO_4_)_3_/S-g-C_3_N_4_ modified glassy carbon electrode

Prior to the modification, the electrode surface was first washed with deionised water and air-dried. For fabrication of the CAP electrochemical sensor, a 1:1 mixture of Gd_2_(WO_4_)_3_/S-g-C_3_N_4_ nanocomposite was first dispersed in 1 mL of deionised water and 6μL of this dispersion was uniformly spread over the GCE surface. The so-modified GCE was dried at room temperature (Scheme 1B) and subsequently, the electrochemical performance of the electrode towards the CAP reduction was investigated.

## Results and discussion

### Characterization of synthesized Gd_2_(WO_4_)_3_/S-g-C_3_N_4_ nanoparticles

**Figure 1.**
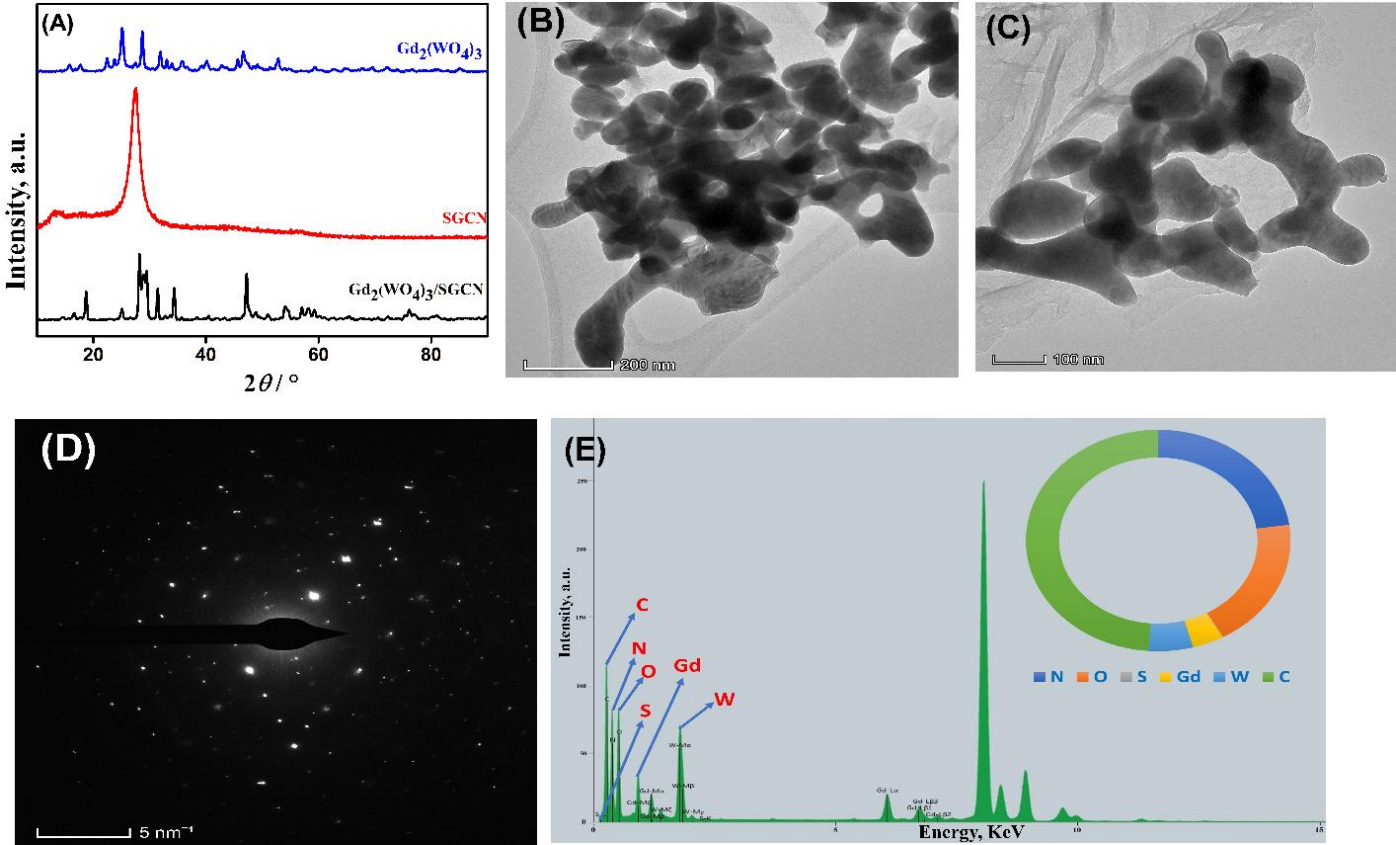
(A) XRD Patterns of Gd_2_(WO_4_)_3_, S-g-C_3_N_4_ and Gd_2_(WO_4_)_3_/S-g-C_3_N_4_, (B) and (C) TEM images of Gd_2_(WO_4_)_3_/S-g-C_3_N_4_, (D) SEAD pattern of Gd_2_(WO_4_)_3_/S-g-C_3_N_4_ and (E) EDX spectrum of nanocomposite with corresponding content, wt.%

X-ray powder diffraction analysis (XRD), which reveals the crystallinity and purity of compounds, was used to examine the synthesised nanocomposite. As can be witnessed from the results obtained in Figure 1A, XRD confirmed the crystal structure of the Gd_2_(WO_4_)_3_ and the sharp scattering peaks could be well indexed to the tetragonal phase (JCPDS No. 25-0829) of pure Gd_2_(WO_4_)_3_ [20]. The 18.73, 25.11, 27.94, 29.50, 31.49, 34.47 and 47.22° are the diffraction peaks and (111), (112), (221), (023), (040), (204) and (242) are the crystal lattices, respectively. Similarly, S-g-C_3_N_4_ exhibited a characteristic XRD peak, which is in excellent agreement with the literature [21,22]. As shown in the graph for the composite of Gd_2_(WO_4_)_3_ and S-g-C_3_N_4_, the crystalline peaks are well matched.

The morphological structure of Gd_2_(WO_4_)_3_/S-g-C_3_N_4_ nanocomposite was examined by TEM. As shown in Figure 1B, the S-g-C_3_N_4_ had a nanosheet-like structure and had a few black patches on or between the surfaces, indicating that elemental sulphur is spread randomly on the surface of the GCN nanosheets. The surface morphology of Gd_2_(WO_4_)_3_ was also recorded as illustrated in Figure 1C, which clearly showed the appearance of a dicot seed-like structure of Gd_2_(WO_4_)_3_ [23,24]. Furthermore, the composite's overall morphology indicates a uniform distribution of materials, free of impurities, and a smooth, flawless surface. This validates the successful synthesis of a nanocomposite.

The SAED pattern of the Gd_2_(WO_4_)_3_/S-g-C_3_N_4_ nanocomposite was captured as depicted in Figure 1D. It displays a pattern of spots that can be related to the planes of (112) and (221), which correlates with the XRD patterns. The elemental distribution was carried out through the EDS that confirmed the presence of all the elements which were used during the synthesis, *i.e.* nitrogen (22.7 %) oxygen (19.1 %) carbon (48.8 %) gadolinium (3.84 %), tungstate (5.43 %) and sulphur (0.0784 %) suggesting the successful synthesis Gd_2_(WO_4_)_3_/S-g-C_3_N_4_ nanocomposite without any notable contaminants.

**Figure 2.**
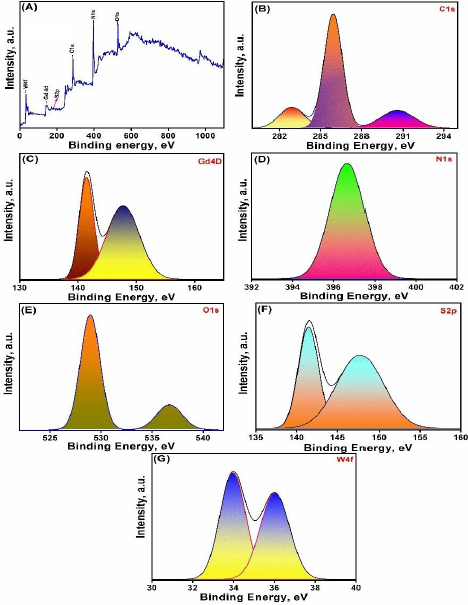
XPS spectra of Gd_2_(WO_4_)_3_/S-g-C_3_N_4_ nanocomposite (A) the survey scan, (B) C 1s, (C) Gd 4D, (D)N 1s, (E) O 1s, (F) S 2p and (G) W 4f

The XPS spectra of the as-synthesised nanocomposite Gd_2_(WO_4_)_3_/S-g-C_3_N_4_ were evaluated to determine the chemical state and elemental composition of materials, as illustrated in Figure 2. In the spectral peaks of C 1s, several peaks are prominent that correspond to several carbon functional groups, including C-C/C-H, C-O, C=O, and COOH. These peaks' relative intensities range from 282 to 290 eV and the locations reveal details about the chemistry of the surface. Whereas, in the N1s XPS spectrum, unique peaks of different nitrogen species, including amine (N-H), amide (C-N), and nitro (N=O) groups, are exhibited. Likewise, peaks from several oxygen species, including metal oxide (M-O), hydroxyl (O-H), and carbonyl (C=O) groups, may be observed in the O 1s XPS spectrum in the 528.9-536.7 eV range. The interpretation of oxidation state is aided by these peaks. The XPS spectra of the S 2p peaks at 141.4 and 147.6 eV are associated with sulphur species, including thiol (S-H), sulphide (S-S), and sulphate (S-O) groups. The core-level peaks corresponding to the Gd and W elements were revealed by the XPS spectra for the Gd4d (141.52 and 147.6 eV) and W4f (33.94 and 36 eV) elements, respectively. These spectra made it possible to identify the oxidation states and chemical surroundings of Gd and W atoms, which are essential for comprehending how they impact the material's distinctive characteristics.

### Electrochemical characterization of Gd_2_(WO_4_)_3_/S-g-C_3_N_4_ modified glassy carbon electrode

#### Surface characterization of the modified electrode using cyclic voltammetry

**Figure 3.**
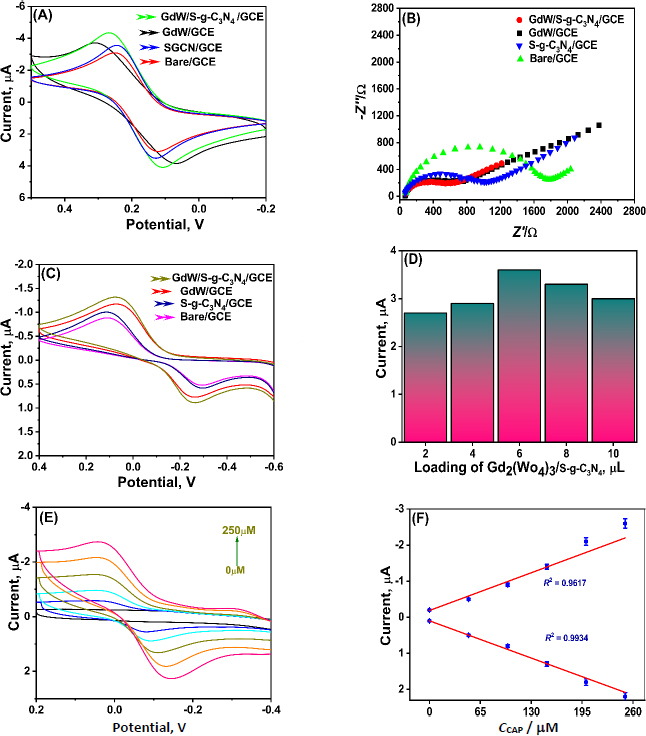
(A) Cyclic voltammograms of bare GCE, S-g-C_3_N_4_/GCE, Gd_2_(WO_4_)_3_/GCE, and Gd_2_(WO_4_)_3_/S-g-C_3_N_4_/GCE electrodes recorded in 5 mM [Fe(CN)_6_]^3-^/^4-^ containing 0.1 M KCl at a scan rate of 50 mV s^-1^; (B) Nyquist plots (EIS) of the corresponding electrodes in 5 mM [Fe(CN)_6_]^3-^/^4-^ with 0.1 M KCl, recorded over a frequency range of 0.1 Hz to 100 kHz at an amplitude of 10 mV; (C) Cyclic voltammograms of individual electrodes (bare GCE, S-g-C_3_N_4_/GCE, Gd_2_(WO_4_)_3_/GCE, and Gd_2_(WO_4_)_3_/S-g-C_3_N_4_/GCE) toward 100 μM CAP in 0.1 M PBS (pH 7.0);(D) Optimization of Gd_2_(WO_4_)_3_/S-g-C_3_N_4_ loading on GCE and corresponding current responses toward 100 μM CAP; (E) Cyclic voltammograms of Gd_2_(WO_4_)_3_/S-g-C_3_N_4_/GCE at varying CAP concentrations (50 to 250 μM) in 0.1 M PBS (pH 7.0); (F) Calibration plots showing the linear relationship between peak current and CAP concentration derived from Figure 3E

Figure 3A shows the voltametric response of Gd_2_(WO_4_)_3_/S-g-C_3_N_4_, Gd_2_(WO_4_)_3_, S-g-C_3_N_4_ and unmodified bare GCE in 5 μM of [Fe (CN)_6_]^3-/4-^ as redox probe in the presence of 0.1 M KCl. Apparently, the bare GCE shows a weak redox peak response with a peak current of 3.06 μA, compared with other modifications such as S-g-C_3_N_4_/GCE and Gd_2_(WO_4_)_3_/GCE, which showed peak responses of 3.53 and 3.86 μA, respectively. Compared with the Gd_2_(WO_4_)_3_/S-g-C_3_N_4_/GCE revealed a very high current response of 4.14 μA. This increase is significantly higher than that for the other modified electrodes. These observations demonstrate the synergistic effects of the nanocomposite, which contribute to its large surface-to-volume ratio and enhanced performance.

The electrochemical sensing mechanism of CAP at the Gd_2_(WO_4_)_3_/S-g-C_3_N_4_ modified electrode is primarily governed by the synergistic interaction between Gd_2_(WO_4_)_3_ and sulphur-doped graphitic carbon nitride. The incorporation of Gd_2_(WO_4_)_3_ onto the S-g-C_3_N_4_ matrix increases the density of catalytically active sites and facilitates faster charge transfer between the electrode surface and the electrolyte. sulphur doping introduces defect sites and enhances the electrical conductivity of g-C_3_N_4_, thereby promoting electron mobility and improving the adsorption affinity of CAP molecules.

Upon applying a suitable potential, the nitro group (-NO_2_) of CAP undergoes a two-step, four-electron reduction process. In the first step, CAP is reduced to the corresponding hydroxylamine derivative (-NHOH), followed by its further reduction to the amine (-NH_2_) form. This reduction process is facilitated by the excellent redox mediation properties of Gd^3+^/Gd^2+^ centres present in Gd_2_(WO_4_)_3_ and the strong π-π interaction between the aromatic ring of CAP and the conjugated g-C_3_N_4_ framework. The presence of sulphur atoms within the g-C_3_N_4_ lattice increases the local electron density near the conduction band, thereby enhancing electron transfer kinetics during the CAP reduction.

Electrochemical impedance spectroscopy (EIS) and cyclic voltammetry (CV) studies further support this mechanism. A significant decrease in charge-transfer resistance and an increased redox current response was observed for the Gd_2_(WO_4_)_3_/S-g-C_3_N_4_ modified electrode compared to pristine g-C_3_N_4_ or bare GCE, confirming the enhanced electrocatalytic efficiency of the composite. The synergistic effect between Gd_2_(WO_4_)_3_ and S-g-C_3_N_4_ results in improved sensitivity, lower detection limits, and superior stability of the sensor toward CAP detection.

**Figure 4.**
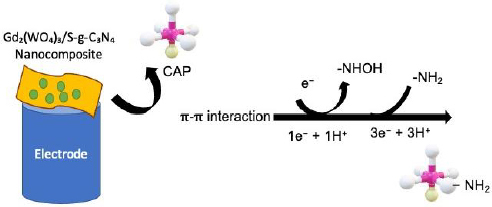
Proposed electrochemical sensing mechanism of CAP at the Gd_2_(WO_4_)_3_/S-g-C_3_N_4_ modified electrode

Figure 4 illustrates the synergistic role of Gd_2_(WO_4_)_3_ and sulphur-doped g-C_3_N_4_ in facilitating the electrochemical reduction of CAP. The S-g-C_3_N_4_ nanosheets provide a high surface area with abundant active sites and improved conductivity, owing to sulphur-induced defects, enabling efficient adsorption of CAP molecules via π-π stacking interactions. The Gd_2_(WO_4_)_3_ nanoparticles anchored on the S-g-C_3_N_4_ surface act as redox mediators, accelerating electron transfer between CAP and the electrode interface. The nitro group (-NO_2_) of CAP is reduced sequentially to the hydroxylamine (-NHOH) and amine (-NH_2_) intermediates via a two-step, four-electron transfer process. This synergistic interplay between Gd_2_(WO_4_)_3_ and S-g-C_3_N_4_ significantly enhances the current response, resulting in high sensitivity and selectivity for CAP detection.

#### Electrochemical impedance spectroscopy

EIS is a highly sensitive technique for evaluating electrode surface characteristics during successive modification steps. In this study, EIS measurements were performed for both modified and unmodified electrodes in the presence of 5 mM [Fe(CN)_6_]^3-^/^4-^ as a redox probe in 0.1 M KCl. The bare GCE displayed a high charge transfer resistance (*R*’) of approximately 1325 Ω, indicating sluggish electron transfer. Upon modification with S-g-C_3_N_4_, the *R*_ct_ decreased to 1038 Ω, while the Gd_2_(WO_4_)_3_-modified electrode exhibited a further reduction to 789 Ω. Notably, the Gd_2_(WO_4_)_3_/S-g-C_3_N_4_-modified GCE showed a much lower *R*_ct_ of 565 Ω, confirming the synergistic interaction between Gd_2_(WO_4_)_3_ and S-g-C_3_N_4_. This enhanced conductivity arises from improved charge-transfer pathways, thereby increasing the electroactive surface area provided by the nanocomposite film.

EIS was performed at the open-circuit potential (OCP) after the electrode had equilibrated for 300 s. The AC perturbation amplitude was 5 mV (rms) and the frequency range spanned 100 kHz to 0.01 Hz (logarithmic frequency sampling). Each spectrum represents the average of three independent measurements (*I* = 3). Nyquist plots show the real part *Z*′ / Ω on the x-axis and the negative imaginary part -*Z*″ / Ω on the y-axis; in the revised figures, the x- and y-axes use the same scale to avoid visual distortion of the semicircle.

The charge-transfer resistance (*R*_ct_) was obtained by fitting the Nyquist spectra to the equivalent circuit model *R*_s_ + *R*_ct_ CPE + *WR* (where *R_s_* is the solution resistance, CPE is the constant phase element representing the double-layer behaviour, and *W* is the Warburg element for diffusion). Curve-fitting was performed using Z View (or the CHI software fitting routine) [25], and the reported *R*_ct_ values correspond to the fitted semicircle diameter (difference between the high-frequency and low-frequency intercepts on the real axis). The fitting quality was assessed by the chi-squared statistic (*χ*^2^ < 10^-3^) and by visual agreement between the experimental and fitted data. Note that *R*_ct_ is reported in Ω; conductance (*G*) is the reciprocal (*G* = 1/*R*) and was not used in the present analysis.

The electroactive surface area (*A*) was calculated using the Randles-Ševčik equation:



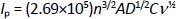

(1)


where *I*_p_ / A is the peak current, *n* = 1, *D* = 7.6×10^-6^ cm^2^ s^-1^, *C* = 5 mM and *ν* = 0.05 V s^-1^ [21]. The calculated surface areas were 0.031 cm^2^ for bare GCE, 0.045 cm^2^ for S-g-C_3_N_4_/GCE, 0.052 cm^2^ for Gd_2_(WO_4_)_3_/GCE, and 0.071 cm^2^ for Gd_2_(WO_4_)_3_/S-g-C_3_N_4_/GCE. The increase in electroactive surface area clearly demonstrates enhanced charge transfer and a higher density of catalytic sites on the composite-modified electrode, contributing to its improved electrochemical performance.

#### Electrochemical activity towards CAP with individual materials.

The electrochemical behaviour of the sensor towards CAP detection was examined by CV in PBS (0.1 M, pH 7.0) in the presence of 100 μM CAP (Figure 3C). Compared to the responses of bare GCE, the response obtained for S-g-C_3_N_4_/GCE, Gd_2_(WO_4_)_3_/GCE as well as Gd_2_(WO_4_)_3_/S-g-C_3_N_4_/GCE electrode was 0.5, 0.59, 0.77 and 0.89 μA, respectively. Likewise, the current response recorded for Gd_2_(WO_4_)_3_/S-g-C_3_N_4_/GCE was higher and actively good. This demonstrated the improved performance of the electrode towards CAP owing to the high surface area and excellent electron conductivity of the synergistic nanocomposite.

#### Optimization of electrode parameters

Different volumes of Gd_2_(WO_4_)_3_:S-g-C_3_N_4_ nanocomposite prepared using an equal weight ratio, *i.e.* 2.5:2.5 of 5 mg of the nanocomposite dispersed in 1 ml of water, were explored for the construction of the sensor to optimise the electrode fabrication protocol. The nanocomposite dispersion was then drop-casted on the GCE electrode surface in varying volumes like 2, 4, 6, 8 and 10 μL. Following this, these electrodes were examined for the electrochemical determination of 100 μL of CAP under standard conditions. As shown in Figure 3D, the current response for CAP was excellent and an enhanced peak was obtained in the case of 6 μL of the nanocomposite. Hence, for our studies throughout this work, 6 μL was selected as a standard volume in the fabrication procedure.

### Electrochemical activity of Gd_2_(WO_4_)_3_/S-g-C_3_N_4_/GCE towards CAP

#### Electrochemical detection of CAP through cyclic voltammetry by increasing CAP concentration

CAP detection was performed using CV techniques. The study was conducted by increasing the concentration range of CAP from 50 to 250 μM in the potential range of -0.4 to 0.2 V at a scan rate (*ν*) of 50 mV s^-1^ in 0.1 M PBS (pH 7.0). The outcome is shown in Figure 3E, and the calibration plot for CAP is shown in the inset. It is evident that an increase in CAP results in higher anodic and cathodic currents. Furthermore, Figure 3F illustrates how the concentration of CAP and the current are interdependent with satisfactory regression coefficient values of *R*^2^= 0.99342 and *R*^2^ = 0.96172 for *I*_pa_ and *I*_ca_, respectively.

#### Effect of pH

**Figure 5.**
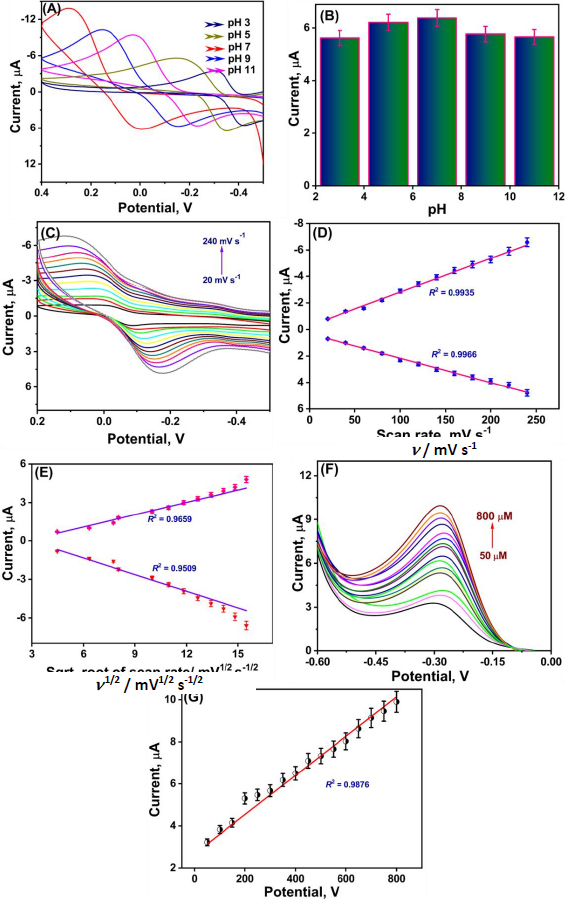
(A) CV response for CAP at different pH (3 to 11); (B) bar graph of pH vs current; (C) effect of scan rate from 20 to 200 mV s^-1^ in presence of 100 mM of CAP in 0.1 M PBS buffer solution (D) linear plot of scan rate *vs.* peak current; (E) linear plot of square root of scan rate *vs.* current; (F) LSV measurements conducted with gradual increasing concentration of CAP from 50 to 800 μM. (G) Linear plot of LSV showing CAP concentrations plotted with current

pH greatly affects the performance of any electrochemical sensor. To achieve a proper electrochemical response, it is necessary to study the effect of pH on the modified electrochemical sensor. The impact of pH on the Gd_2_(WO_4_)_3_/S-g-C_3_N_4_ nanocomposite was examined at different pH levels of 3, 5, 7, 9 and 11 in the presence of 100 μL of CAP. As shown in Figure 5A, the CAP redox peak increases with increasing pH from 3 to 7. However, further increases in pH beyond 7 result in a decrease in the peak current, as shown in the bar graph (Figure 5B). The highest peak current is at pH 7. The inset bar graph represents the peak current responses to different pH solutions. To increase the electrochemical sensor's sensitivity, pH 7 is used for all experiments.

#### Effect of scan rate

The relationship between scan rate and redox current was investigated to understand the electron-transport process from the bulk solution to the electrode surface. At scan rates ranging from 20 to 240 mV s-1, CV was recorded for 100 mM CAP in 0.1 M PBS buffer. The results are shown in Figure 5C.

The anodic and cathodic peak currents were found to increase as the scan rate was gradually increased. Figure 5D displays the corresponding linear plot of the anodic and cathodic peak currents versus the scan rates. In addition, the square root of the scan rate was also plotted and is shown in Figure 5E. The obtained peak currents followed the linear relationship *I*_pa_ = *R*^2^ = 0.9962 and *I*_ca_ = *R*^2^ = 0.9935 with the scan rates in the investigated range. The calibration plot was drawn to determine the linearity of the response, and from the obtained result, we can clearly observe linearity based on the *R*^2^ Value, which is almost 1. From these plots, we can confirm that the electrode process is adsorption-controlled.

Optimizing voltammetric parameters is crucial for achieving the best analytical performance of the modified electrode. Parameters such as pH, scan rate, and nanocomposite loading volume were systematically varied. The highest current response was obtained at pH 7.0, confirming that near-neutral conditions favour efficient electron transfer for CAP reduction. Similarly, the scan rate study (20 to 240 mV s^-1^) revealed a linear increase in current with the square root of the scan rate, indicating an adsorption-controlled process. Among various loading volumes, 6 μL of Gd_2_(WO_4_)_3_/S-g-C_3_N_4_ dispersion yielded the maximum current response; hence, these optimized parameters were adopted for subsequent analyses.

#### Electrochemical detection of CAP by using linear sweep voltammetry

To further validate the enhanced electrochemical performance of the proposed electrode, linear sweep voltammetry (LSV) investigation was conducted. the sensing capabilities of the prepared Gd_2_(WO_4_)_3_/S-g-C_3_N_4_ electrocatalyst towards CAP quantitation in the presence of varying concentrations of CAP in 0.1 M PBS solution at a constant scan rate of 50 mV s^-1^ were evaluated. Figure 5F shows that the oxidation current gradually increases with the addition of CAP, one at a time, into the reaction medium, indicating the electrocatalyst's sensing effectiveness.

Additionally, a linear relationship between peak current and CAP concentration was observed over the entire range of 50-800 μM (Figure 5G), with a calibration equation *R*^2^ = 0.98766. The sensitivity of the proposed sensor was calculated by dividing the slope value by the electrode surface area (0.071 cm^2^), Equation (2):



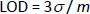

(2)


where *m* is the slope of the current *vs.* concentration curve and *σ* is the standard deviation of three measurements of blank (0.1 M PBS electrolyte without analyte). The LOD value determined using Equation (2) was found to be 49.143 nM. Similarly, the limit of quantification (LOQ) calculated using Equation (3) was found to be 163.811 nM.



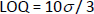

(3)


To validate the overall performance of the electrode, the results were compared with previously published literature and presented in Table 1.

**Table 1:** Comparison of different electrochemical sensors for the determination of CAP

No	Technique	Material	LOD, μM	Reference
1	DPV	Cl-RGO/GCE	1.0	[2]
2	DPV	CoMoO_4_/GCE	0.014	[26]
3	SWV	EPC/GCE	2.9*	[27]
4	DPV	Co-Fe_3_O_4_ NS/GO	0.00104	[28]
5	SWV	Fe_3_O_4_-CMC@Au	0.07	[29]
6	LSV	rGO-Pt-Pd NCs	0.1	[30]
7	DPV	Co_3_O_4_@rgo	1.16	[31]
8	DPV	MoS_2_-IL/GO	0.047	[32]
9	DPV	Fe_3_O_4_/GCE	0.09	[33]
10	LSV	Gd_2_(WO_4_)_3_/S-g-C_3_N_4_/GCE	0.0491	This work

*nmol L^-1^

#### Selectivity

**Figure 6.**
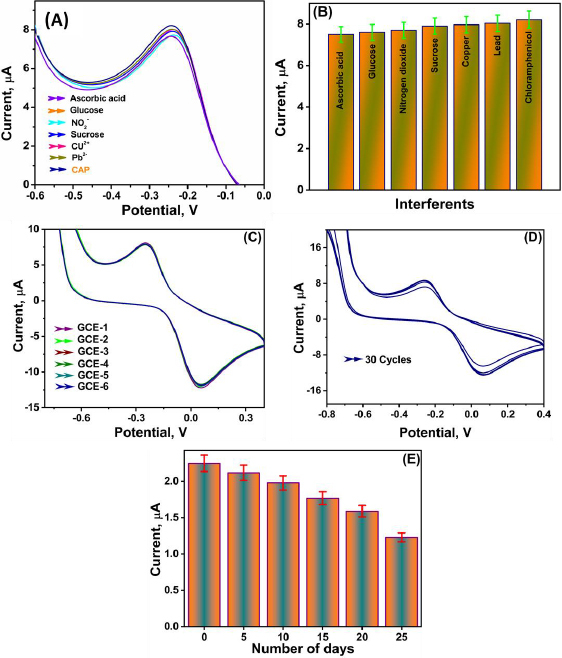
(A) LSV plots for the study of interferents in the presence of ascorbic acid, glucose, nitrogen dioxide, sucrose, copper, and lead along with CAP; (B) bar graph showing the current response of interferents in presence of CAP; (C) CV plots for reproducibility analysis of 6 electrodes; (D) CV plots for study of repeatability- cyclic stability with 30 cycles; (E) storage stability of the sensor from day 1 to day 25

A selectivity test was performed to evaluate the electrocatalytic performance of the developed electrochemical sensor for the selective determination of CAP, as shown in Figure 6A. In this application test, the interferants such as ascorbic acid, glucose, NO_2_, sucrose, Cu^2+^ and Pb^2-^ were taken and individually added along with 3 % higher concentration of CAP under optimized conditions at the scan rate of 50 mV s^-1^. The results obtained confirmed that the nanocomposite-modified GCE was successful in-selectively detecting CAP. This can be correlated with the LSV technique as the-potentials obtained towards CAP determination.

#### Reproducibility, cyclic stability, and storage stability of the sensor

The proposed sensor's practical viability, including reproducibility, cycle stability, and storage stability, was assessed using the CV approach in the presence of 100 μM CAP in 0.1 M PBS (pH 7). To test the reproducibility, five different fabricated using a same protocol, wherein the Gd_2_(WO_4_)_3_/S-g-C_3_N_4_ nanocomposite was drop casted on GCE electrodes were used for CAP determination (Figure. 6C). The extraordinary repeatability of Gd_2_(WO_4_)_3_/S-g-C_3_N_4_/GCE is illustrated by the fact that the redox peaks of five different electrodes differed only by less than 5 % of the actual electrode.

Similarly, Figure 6D illustrates the stability of the modified electrodes, assessed using 30 repetitive potential cycles. The cyclic voltammograms could be repeated without significantly altering the background current response. It is observed that after 30 cycles, the modified electrode retained excellent stability, with only a negligible decrease in current compared to the second cycle and a minor change from the first cycle, which can be readily neglected.

To carry out the stability studies, modified Gd_2_(WO_4_)_3_/S-g-C_3_N_4_/GCE electrodes were used to determine CAP rinsed with water, and then dried in an ambient atmosphere and stored at 4 °C when not in use. This process of analysis, rinsing and storing was carried out for up to 25 days. The results obtained were then compared with those of a newly fabricated sensor.

As shown in Figure 6E, peak current readings taken for electrodes with a 1 M concentration of CAP were nearly a perfect match for readings taken for a newly prepared electrode. Thus, we can conclude that the developed sensor showed good relative stability over time as the peak current response was 100, 95.45, 86.36, 77.21, 68.18 and 54.54 % for 1, 5, 10, 15, 20 and 25 days, respectively.

#### Real sample analysis

The quantitative analysis of CAP in real samples was done using the LSV method to evaluate its practical applicability. To perform this study, the CAP concentration in a commercially available Eye drop and a milk sample was first determined using the developed electrode. As a result, it was possible to assess how well the new electrode performed while analysing the actual sample data. The outcomes of the recovery trials are shown in Table 2 and demonstrate outstanding recovery and sensor validity in real-world applications.

**Table 2:** Detection of CAP in real samples

Samples	Amount, μM		Recovery, %	RSD, %
	Added	Found		
Milk	25	24.8	99.2	2.7
	50	49.2	98.8	2.2
Eye drops	25	24.9	99.6	3.9
	50	49.95	99.9	2.85

## Conclusion

Here, we use a simple and efficient co-precipitation technique to prepare a Gd_2_(WO_4_)_3_/S-g-C_3_N_4_ nanocomposite, which has potential for a wide range of electrochemical sensing applications. Firstly, Gd_2_(WO_4_)_3_/S-g-C_3_N_4_ is embellished on the GCE in its as-synthesised form. To characterise the Gd_2_(WO_4_)_3_/S-g-C_3_N_4_ nanocomposite, a variety of spectrophotometric methods like TEM, EDX with elemental mapping, XPS and XRD are used. Subsequently, the electrochemical properties of the Gd_2_(WO_4_)_3_/S-g-C_3_N_4_ modified GCE towards the determination of CAP were investigated using CV, EIS and LSV. The LSV revealed interesting results with a very low LOD of 49.143 nM for CAP determination. The synergistic effects of Gd_2_(WO_4_)_3_/S-g-C_3_N_4_ contributed to the excellent electroanalytical characteristics of the proposed sensor. The modified sensor also showed good cyclic stability, storage stability and reproducibility along with an excellent anti-interfering activity.

## References

[1] Sun T. Pan H. Mei Y. Zhang P. Zeng D. Liu X. Rong S. Chang D. Electrochemical sensor sensitive detection of chloramphenicol based on ionic-liquid-assisted synthesis of de-layered molybdenum disulfide/graphene oxide nanocomposites. Journal of Applied Electrochemistry 49(3) (2019) 261-270. https://doi.org/10.1007/s10800-018-1271-6 10.1007/s10800-018-1271-6

[2] Wang K.P. Zhang Y.C. Zhang X. Shen L. Green preparation of chlorine-doped graphene and its application in electrochemical sensor for chloramphenicol detection. SN Applied Sciences 1(2) (2019) 157. https://doi.org/10.1007/s42452-019-0174-4 10.1007/s42452-019-0174-4

[3] Gao S. Zhang Y. Yang Z. Fei T. Liu S. Zhang T. Electrochemical chloramphenicol sensors based on trace MoS_2_ modified carbon nanomaterials: Insight into carbon supports. Journal of Alloys and Compounds 872 (2021) 159687. https://doi.org/10.1016/j.jallcom.2021.159687 10.1016/j.jallcom.2021.159687

[4] Sebastian N. Yu W.C. Balram D. Electrochemical detection of an antibiotic drug chloramphenicol based on a graphene oxide/hierarchical zinc oxide nanocomposite. Inorganic Chemistry Frontiers 6(1) (2019) 82-93. https://doi.org/10.1039/C8QI01000E 10.1039/C8QI01000E

[5] Shad A.N. Bajwa S.Z. Amin N. Taj A. Hameed S. Khan Y. Dai Z. Cao C. Khan W. S. Solution growth of 1D zinc tungstate (ZnWO_4_) nanowires; design, morphology, and electrochemical sensor fabrication for selective detection of chloramphenicol. Journal of Hazardous Materials 367 (2019) 205-214. https://doi.org/10.1016/j.jhazmat.2018.12.072 10.1016/j.jhazmat.2018.12.072 30594721

[6] Yadav M. Ganesan V. Gupta R. Yadav D.K. Sonkar P.K. Cobalt oxide nanocrystals anchored on graphene sheets for electrochemical determination of chloramphenicol. Microchemical Journal 146 (2019) 881-887. https://doi.org/10.1016/j.microc.2019.02.025 10.1016/j.microc.2019.02.025

[7] Selvi S.V. Nataraj N. The electro-catalytic activity of nanosphere strontium doped zinc oxide with rGO layers screen-printed carbon electrode for the sensing of chloramphenicol. Microchemical Journal 159 (2020) 105580. https://doi.org/10.1016/j.microc.2020.105580. 10.1016/j.microc.2020.105580

[8] Jaysiva G. Manavalan S. Chen S.M. Veerakumar P. Keerthi M. Tu H.S. MoN nanorod/sulfur-doped graphitic carbon nitride for electrochemical determination of chloramphenicol. ACS Sustainable Chemistry & Engineering 8(30) (2020) 11088-11098. https://doi.org/10.1021/acssuschemeng.0c00502 10.1021/acssuschemeng.0c00502

[9] Karuppiah C. Venkatesh K. Hsu L.F. Arunachalam P. Yang C.C. Ramaraj S.K. Ramalingam R.J. Arokiyaraj S. Al-Lohedan H.A. An improving aqueous dispersion of polydopamine functionalized vapor grown carbon fiber for the effective sensing electrode fabrication to chloramphenicol drug detection in food samples. Microchemical Journal 170 (2021) 106675. https://doi.org/10.1016/j.microc.2021.106675 10.1016/j.microc.2021.106675

[10] Akter Mou S. Islam R. Shoeb M. Nahar N. Determination of chloramphenicol in meat samples using liquid chromatography-tandem mass spectrometry. Food Science & Nutrition 9(10) (2021) 5670-5675. https://doi.org/10.1002/fsn3.2530 10.1002/fsn3.2530 34646535 PMC8497835

[11] Vilian A.E. Oh S.Y. Rethinasabapathy M. Umapathi R. Hwang S.K. Oh C.W. Park B. Huh Y.S. Han Y.K. Improved conductivity of flower-like MnWO_4_ on defect engineered graphitic carbon nitride as an efficient electrocatalyst for ultrasensitive sensing of chloramphenicol. Journal of Hazardous Materials 399 (2020) 122868. https://doi.org/10.1016/j.jhazmat.2020.122868 10.1016/j.jhazmat.2020.122868 32531674

[12] Baikeli Y. Mamat X. He F. Xin X. Li Y. Aisa H. A. Hu G. Electrochemical determination of chloramphenicol and metronidazole by using a glassy carbon electrode modified with iron, nitrogen co-doped nanoporous carbon derived from a metal-organic framework (type Fe/ZIF-8). Ecotoxicology and Environmental Safety 204 (2020) 111066. https://doi.org/10.1016/j.ecoenv.2020.111066 10.1016/j.ecoenv.2020.111066 32781344

[13] Xia Y.M. Zhang W. Li M. Y. Xia M. Zou L. J. Gao W. W. Effective electrochemical determination of chloramphenicol and florfenicol based on graphene/copper phthalocyanine nanocomposites modified glassy carbon electrode. Journal of The Electrochemical Society 166(8) (2019) B654. https://iopscience.iop.org/article/10.1149/2.0801908jes/meta

[14] Kokulnathan T. Chen S.M. Design and construction of the gadolinium oxide nanorod-embedded graphene aerogel: a potential application for electrochemical detection of postharvest fungicide. ACS Applied Materials & Interfaces 12(14) (2020) 16216-16226. https://doi.org/10.1021/acsami.9b20224. 10.1021/acsami.9b20224 32149501

[15] Ke Y. Sun Y. Lin P. Zhou J. Xu Z. Cao C. Yang Y. Hu S. Quantitative determination of rare earth elements in scheelite via LA-ICP-MS using REE-doped tungstate single crystals as calibration standards. Microchemical Journal 145 (2019) 642-647. https://doi.org/10.1016/j.microc.2018.11.016. 10.1016/j.microc.2018.11.016

[16] Esmaeili C. Karimi M. S. Norouzi P. Wu F. Ganjali M. R. Safitri E. Gadolinium (III) Tungstate Nanoparticles Modified Carbon Paste Electrode for Determination of Progesterone Using FFT Square-Wave Voltammetry Method. Journal of The Electrochemical Society 167(6) (2020) 067513. https://iopscience.iop.org/article/10.1149/1945-7111/ab828e/meta.

[17] Cheng J. Wang M. Preparation and electrical properties of gadolinium-doped strontium tungstate electrolyte for SOFC. Functional Materials Letters 13(03) (2020) 2050010. https://doi.org/10.1142/S1793604720500101. 10.1142/S1793604720500101

[18] Sakthivel A. Chandrasekaran A. Jayakumar S. Manickam P. Alwarappan S. Sulphur doped graphitic carbon nitride as an efficient electrochemical platform for the detection of acetaminophen. Journal of The Electrochemical Society 166(15) (2019) B1461. https://doi.org/10.1149/2.0021915jes 10.1149/2.0021915jes

[19] Vinoth S. Devi K. S. Pandikumar A. A comprehensive review on graphitic carbon nitride based electrochemical and biosensors for environmental and healthcare applications. Trends in Analytical Chemistry 140 (2021) 116-274. https://doi.org/10.1016/j.trac.2021.116274 10.1016/j.trac.2021.116274

[20] Kalidasan K. Mallapur S. Kulkarni B.B. Maradur S.P. Kumar D. Deeksha R. Kandaiah S. Vishwa P. Girish Kumar S. Gadolinium modified g-C_3_N_4_ for S-Scheme heterojunction with monoclinic-WO_3_: Insights from DFT studies and related charge carrier dynamics. Journal of Materials Research and Technology 204 (2025) 166-176. https://doi.org/10.1016/j.jmst.2024.03.017 10.1016/j.jmst.2024.03.017

[21] Zhu R. Zhang Y. Fang X. Cui X. Wang J. Yue C. Fang W. Zhao H. Li Z. In situ sulfur-doped graphitic carbon nitride nanosheets with enhanced electrogenerated chemiluminescence used for sensitive and selective sensing of l-cysteine. Journal of Materials Chemistry B 7(14) (2019) 2320-2329. https://doi.org/10.1039/C9TB00301K 10.1039/C9TB00301K 32254680

[22] Starukh H. Praus P. Doping of graphitic carbon nitride with non-metal elements and its applications in photocatalysis. Catalysts 10(10) (2020) 1119. https://doi.org/10.3390/catal10101119 10.3390/catal10101119

[23] Sakthivel A. Chandrasekaran A. Jayakumar S. Manickam P. Alwarappan S. Sulphur doped graphitic carbon nitride as an efficient electrochemical platform for the detection of acetaminophen. Journal of The Electrochemical Society 166(15) (2019) B1461. https://doi.org/10.1149/2.0021915jes 10.1149/2.0021915jes

[24] Yu X. Aodenggerile Jiang Z. Shen J. Yan Z. Li W. Qiu H. Integrating the second near-infrared fluorescence imaging with clinical techniques for multimodal cancer imaging by neodymium doped gadolinium tungstate nanoparticles. Nano Research 14 (2021) 2160-2170. https://doi.org/10.1007/s12274-020-3136-7. 10.1007/s12274-020-3136-7

[25] Ravikumar S.B. Mallu T.A. Subbareddy S. Shivamurthy S.A. Neelalochana V.D. Shantakumar K.C. Rajabathar J.R. Ataollahi N. Shadakshari S. An enhanced non-enzymatic electrochemical sensor based on the Bi_2_S_3_-TiO_2_ nanocomposite with HNTs for the individual and simultaneous detection of 4-nitrophenol and nitrofurantoin in environmental samples. Journal of Materials Chemistry B 12(36) (2024) 9005-9017. https://doi.org/10.1039/D3TB03054G 10.1039/D3TB03054G 39149933

[26] Vinothkumar V. Abinaya M. Chen S.M. Ultrasonic assisted preparation of CoMoO_4_ nanoparticles modified electrochemical sensor for chloramphenicol determination. Journal of Solid State Chemistry 302 (2021) 122392. https://doi.org/10.1016/j.jssc.2021.122392 10.1016/j.jssc.2021.122392

[27] Xiao L. Xu R. Yuan Q. Wang F. Highly sensitive electrochemical sensor for chloramphenicol based on MOF derived exfoliated porous carbon. Talanta 167 (2017) 39-43. https://doi.org/10.1016/j.talanta.2017.01.078 10.1016/j.talanta.2017.01.078 28340736

[28] Nehru R. Dong C. D. Chen C.W. Cobalt-doped Fe_3_O_4_ nanospheres deposited on graphene oxide as electrode materials for electrochemical sensing of the antibiotic drug. ACS Applied Nano Materials 4(7) (2021) 6768-6777. https://doi.org/10.1021/acsanm.1c00826. 10.1021/acsanm.1c00826

[29] Jakubec P. Urbanová V. Medříková Z. Zbořil R. Advanced sensing of antibiotics with magnetic gold nanocomposite: Electrochemical detection of chloramphenicol. Chemistry - A European Journal 22(40) (2016) 14279-14284. https://doi.org/10.1002/chem.201602434 10.1002/chem.201602434 27529758

[30] Kong F.Y. Luo Y. Zhang J.W. Wang J.Y. Li W.W. Wang W. Facile synthesis of reduced graphene oxide supported Pt-Pd nanocubes with enhanced electrocatalytic activity for chloramphenicol determination. Journal of Electroanalytical Chemistry 781 (2016) 389-394. https://doi.org/10.1016/j.jelechem.2016.06.018 10.1016/j.jelechem.2016.06.018

[31] Yadav M. Ganesan V. Gupta R. Yadav D.K. Sonkar P.K. Cobalt oxide nanocrystals anchored on graphene sheets for electrochemical determination of chloramphenicol. Microchemical Journal 146 (2019) 881-887. https://doi.org/10.1016/j.microc.2019.02.025 10.1016/j.microc.2019.02.025

[32] Sun T. Pan H. Mei Y. Zhang P. Zeng D. Liu X. Rong S. Chang D. Electrochemical sensor sensitive detection of chloramphenicol based on ionic-liquid-assisted synthesis of de-layered molybdenum disulfide/graphene oxide nanocomposites. Journal of Applied Electrochemistry 49(3) (2019) 261-270. https://doi.org/10.1007/s10800-018-1271-6 10.1007/s10800-018-1271-6

[33] Giribabu K. Jang S.C. Haldorai Y. Rethinasabapathy M. Oh S.Y. Rengaraj A. Han Y.K. Cho W.S. Roh C. Huh Y.S. Electrochemical determination of chloramphenicol using a glassy carbon electrode modified with dendrite-like Fe_3_O_4_ nanoparticles. Carbon Letters 23 (2017) 38-47. https://doi.org/10.5714/CL.2017.23.038 10.5714/CL.2017.23.038

